# Preventive Gambling Programs for Adolescents and Young Adults: A Systematic Review

**DOI:** 10.3390/ijerph20064691

**Published:** 2023-03-07

**Authors:** Alicia Monreal-Bartolomé, Alberto Barceló-Soler, Javier García-Campayo, Cruz Bartolomé-Moreno, Paula Cortés-Montávez, Esther Acon, María Huertes, Víctor Lacasa, Sofía Crespo, Daniel Lloret-Irles, Luis Sordo, Catrina Clotas Bote, Susanna Puigcorbé, Yolanda López-Del-Hoyo

**Affiliations:** 1 Institute of Health Research of Aragon (IIS Aragón), Miguel Servet University Hospital, 50009 Zaragoza, Spain; 2 Chronicity, Primary Care, and Health Promotion Research Network (RICAPPS), 50015 Zaragoza, Spain; 3 Department of Psychology and Sociology, University of Zaragoza, 50009 Zaragoza, Spain; 4 Healthcare Research Institute of Navarre (IdiSNA), 31008 Pamplona, Spain; 5 Department of Psychiatry, Hospital Miguel Servet, University of Zaragoza, 50009 Zaragoza, Spain; 6 Primary Care Teaching Unit for Zaragoza Healthcare District 1, 50018 Zaragoza, Spain; 7 Department of Health Psychology, Miguel Hernández University, 03202 Elche, Spain; 8 Department of Public Health and Maternal and Child Health, Faculty of Medicine, Complutense University of Madrid, 28040 Madrid, Spain; 9 CIBER Epidemiología y Salud Pública (CIBERESP), 28029 Madrid, Spain; 10 Barcelona Public Health Agency (ASPB), 08023 Barcelona, Spain; 11 Public Health Agency of Catalonia (ASPCAT), 08005 Barcelona, Spain

**Keywords:** prevention, gambling disorder, problem gambling, youth, adolescents

## Abstract

Gambling disorder in youth is an emerging public health problem, with adolescents and young adults constituting a vulnerable age group for the development of gambling-related problems. Although research has been conducted on the risk factors for gambling disorder, very few rigorous studies can be found on the efficacy of preventive interventions in young people. The aim of this study was to provide best practice recommendations for the prevention of disordered gambling in adolescents and young adults. We reviewed and synthesized the results of existing RCTs and quasi-experimental studies covering nonpharmacological prevention programs for gambling disorder in young adults and adolescents. We applied the PRISMA 2020 statement and guidelines to identify 1483 studies, of which 32 were included in the systematic review. All studies targeted the educational setting, i.e., high school and university students. Most studies followed a universal prevention strategy, that particularly targeted adolescents, and an indicated prevention strategy for university students. The reviewed gambling prevention programs generally showed good results in terms of reducing the frequency and severity of gambling, and also regarding cognitive variables, such as misconceptions, fallacies, knowledge, and attitudes towards gambling. Finally, we highlight the need to develop more comprehensive prevention programs that incorporate rigorous methodological and assessment procedures before they are widely implemented and disseminated.

## 1. Introduction

Gambling has become an increasingly normalized, accessible (thanks to the internet and liberalization of gambling regulation), and recreational activity [1]. It is estimated that between 0.1 and 3.4% of the population of Europe, and between 0.1 and 5.8% of the worldwide population engage in problem gambling behavior [2]. Additionally, in 2020, 64.2% of the Spanish population admitted to having gambled at least once in the last 12 months [3]. These percentages have grown over the years, partly explained by the rise in new forms of internet gambling, particularly among young people [3] such as microtransactions or purchases made within video games and sports betting [4], and the direct effect of advertising that typically presents gambling as an interesting, socially desirable, and fun activity [5,6,7], allowing “easy money” to be made [8]. This makes it extremely attractive to adolescents and young adults, with a very limited or nonexistent income.

The number of at-risk gamblers (i.e., individuals who gamble frequently but who have not reached the level of pathological gamblers) has been increasing over the years [9]. In this regard, there are four types of gamblers [10,11]: social and professional gamblers (their gambling does not affect any of the areas of their life), problem gamblers (gambling affects certain aspects of their life without meeting clinical criteria), and pathological gamblers (their thoughts and actions only revolve around gambling, affecting all or most areas of their life; i.e., they meet the criteria for a clinical diagnosis [12]).

Consequently, gambling disorder, in both young people and adults, can be considered on a continuum of gambling behaviors ranging from no gambling of any kind, to social, occasional, or recreational gambling, and problem gambling, to pathological gambling or gambling disorder [13,14]. Furthermore, it is an important clinical problem associated with reduced quality of life, psychiatric comorbidity, cognitive deficits, and a higher risk of suicide [15,16,17]. Therefore, because of its harmful psychosocial, behavioral, economic, academic, occupational, interpersonal, family, mental health, and legal consequences, it is considered a global public health problem [1,13,18,19,20,21,22].

Epidemiological studies conducted in many countries around the world have shown that the first gambling experiences take place between the ages of 10 and 19 years [11]. Moreover, evidence suggests that a younger age of onset is related to greater severity of gambling problems [23] and that young people (under the age of 30 years) are one of the most common age groups to suffer from gambling disorders [24]. This implies that these ages are key periods in the individuals’ relationship with gambling. What is more, these individuals are at a high risk of finding gambling appealing, mainly owing to their likelihood to consider it an economically advantageous activity, a belief that is also strongly associated with developing gambling disorder [25,26].

In this regard, preventing gambling behaviors in adolescents and young adults becomes particularly important. In the literature, gambling prevention strategies can be classified into three levels depending on the different target population groups [27,28]: (1) universal prevention, which aims to reduce the likelihood of a problem behavior in the general population; (2) selective prevention, which targets specific subgroups of the population at higher risk of developing a problem behavior; and (3) indicated prevention, which addresses individuals who are identified to be at high risk for the behavior before it becomes manifest.

Systematic reviews have been conducted into gambling prevention programs for adolescents [29] and young adults [30]; however, most of the studies have primarily focused on the educational settings of high schools and universities, and were conducted more than 5 years ago, which means that they do not represent the most current evidence or knowledge, particularly regarding online gambling, whose prevalence has grown in recent years, especially in the younger population [3].

In order to provide best practice recommendations for the prevention of pathological gambling among adolescents and young adults, the aim of the present study is to systematically review and synthesize the results of existing studies that deal with nonpharmacological prevention programs for gambling disorder.

## 2. Materials and Methods

### 2.1. Design

A systematic review of quantitative studies, including randomized controlled trials (RCTs) and quasi-experimental studies, was conducted. This systematic review was implemented in accordance with the standards of Preferred Reporting Items for Systematic Reviews and Meta-analysis (PRISMA) guidelines [31,32].

### 2.2. Data Sources and Search Strategy

The search strategy implemented to conduct this systematic review relied on the use of the following databases: Pubmed, Embase, Scopus, Web of Science, ScienceDirect, Cochrane Central Register of Controlled Trials (CENTRAL), and ClinicalTrials.gov. A selection was made from all studies published up to 8 April 2022. 

For this purpose, a set of predefined terms combined with the “AND” Boolean operator was used: (1) gambling terms (gambling, gambling disorder, pathological gambling, problem gambling, gambl*, bet*) with the “OR” Boolean operator; (2) intervention terms (prevention, intervention, program) with the “OR” Boolean operator; (3) study type (RCT, trial, randomized controlled trial, quasi-experimental) with the “OR” Boolean operator.

In addition, a chain search, also known as snowballing sampling, was conducted to find new articles from those already selected [33].

### 2.3. Elegibility Criteria

The following inclusion and exclusion criteria were guided by the evidence-based medicine PICOS framework or strategy [21]:

- Participants: Studies that include a target population whose age ranges between 12 and 25 years were included. On the other hand, studies whose criteria did not specify age or in which the age range of the sample was not indicated were excluded.
- Interventions and comparisons: The prevention programs included in this review were non-pharmacological. These could be individual or group-based and could be conducted either in-person or online. Blended programs (i.e., a combination of online and face-to-face sessions) were also included. The duration of the programs could be any. There were no inclusion/exclusion criteria regarding comparators/controls.
- Outcomes: A wide variety of outcomes were considered in this systematic review, such as frequency of gambling, amount of money gambled, and severity of gambling, changes in cognitive variables (such as misconceptions and gambling-related fallacies), and in skills (such as coping, awareness and self-control, problem solving, and decision making).
- Study design: We included randomized clinical trials (RCTs), quasi-experimental studies, and pre–post studies without control group dealing with gambling prevention programs. Studies on protocolized or clearly described programs and where efficacy or effectiveness has been demonstrated by the presentation of some kind of pre–post intervention were also included, as well as studies that report primary data. On the other hand, documents that do not provide original information, those that only report qualitative data or are not available in English or Spanish, and programs whose information is scarce or difficult to access were excluded.

### 2.4. Search Outcomes

**Figure 1:**
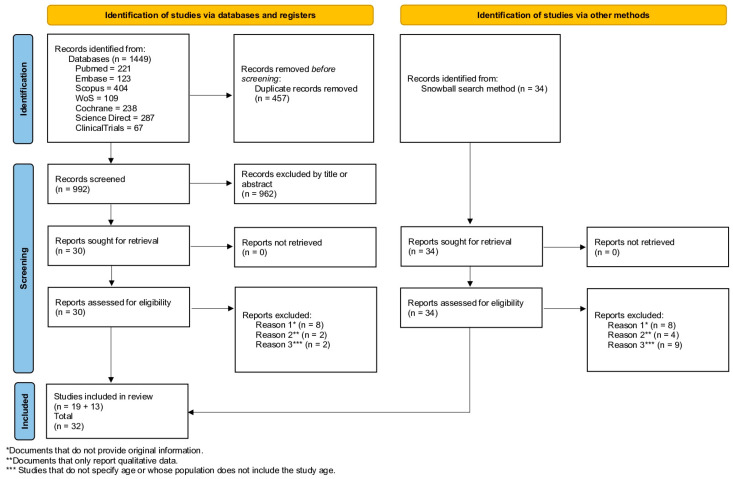
PRISMA diagram of study flow.

Initially, 1449 articles were retrieved from the databases Pubmed, Embase, Scopus, Web of Science, ScienceDirect, Cochrane Central Register of Controlled Trials (CENTRAL), and ClinicalTrials.gov; in addition, 34 articles were identified from the citation search. Once duplicates were removed, a total of 992 articles were identified and screened by three reviewers (P.C.-M., E.A., and M.H.). The full-text manuscripts for 64 studies were then sought for retrieval and assessed, of which 32 met the inclusion criteria (Figure 1).

### 2.5. Quality Appraisal

To assess the quality of the studies, we used the assessment tool developed by the National Heart, Lung, and Blood Institute, one for controlled intervention studies and one for single-arm studies. This allowed the included studies, both controlled intervention studies and single-arm studies, to be evaluated for risk of bias.

Two reviewers (A.M.-B. and E.A.) conducted such a quality assessment of the included studies, and disagreements were resolved through discussion between the researchers and in consultation with a third author (Y.L.-H.). The quality assessment is provided in the Supplementary Materials (Tables S1 and S2) along with a link to the assessment tools and their items.

### 2.6. Data Abstraction

Three independent reviewers took part in the study selection process (P.C.-M., E.A., and M.H.), two of whom individually applied the inclusion and exclusion criteria to assess the eligibility of the studies retrieved from the search (after removal of any duplicates) in two stages: the first based on titles and abstracts, and the second based on the full texts. Any disputes that arose during either stage were resolved by discussion between the two main reviewers and a third reviewer, after which a final decision was made. Figure 1 shows the identified, eliminated, and selected articles during the review process.

Data extraction from the included articles was then performed independently by two other reviewers (A.B.-S. and S.C.). Extracted data included: (1) participants, (2) type of prevention, (3) intervention, (4) comparison, (5) study design, (6) follow-ups, (7) result measurement instruments, and (6) results. 

## 3. Results

### 3.1. Study Characteristics

The first characteristic that can be appreciated is the age of participants. Most of the studies targeted adolescents (75%) over young adults (25%). In the case of studies conducted in university settings, none applied a universal prevention strategy to protect both gamblers and nongamblers from the negative consequences of this activity, and only two studies applied selective prevention [34,35]. The remainder of these studies applied an indicated prevention strategy. In contrast, most of the studies that focused on high school students applied universal prevention strategies, mainly by raising awareness and correcting misperceptions and beliefs, followed by selective strategies; only one study applied an indicated strategy [36]. What is more, all the studies involving university students were conducted in the United States and Canada. In the case of studies involving adolescents, while the settings of the earlier works were mostly the United States and Canada, studies began to be conducted in Europe in 2013, with a larger number of European studies in recent years. Finally, the number of studies conducted on Spanish samples is scarce [37,38,39], and the first was only published in 2020.

In addition, differences were observed in relation to the sample sizes of the different studies included in this systematic review. Consequently, there were both studies involving a small sample size (67, 75, 81 participants in [40,41,42]) and a large sample size (16,421 and 8455 participants in [43,44]). In general, they mostly involved males, with very little difference in percentages. Only the study by Larimer et al., dated 2012 [45], differentiated between participants by origin. Moreover, randomized clinical trials predominated, and most did not include a long-term follow-up (see Table 1).

**Figure 2:**
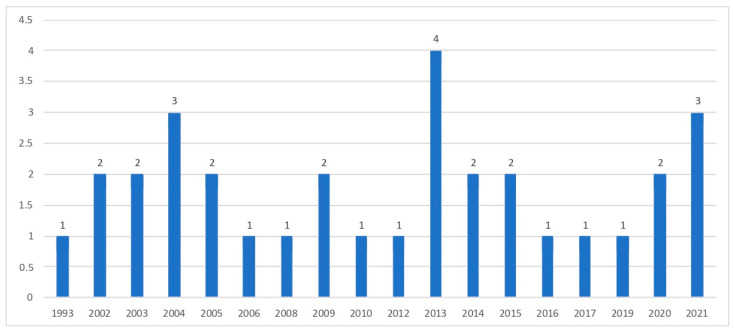
Distribution by year of publication.

Finally, it is important to note the age of the studies, such as that conducted in 1993 by Gaboury and Ladouceur [46], and their variability in terms of the interest generated by these types of study, with different periods of higher and lower numbers published (see Figure 2).

### 3.2. Effects of Programs

In general, in terms of the changes observed in the measured cognitive variables, the programs were effective for reducing erroneous gambling-related ideas and fallacies by increasing knowledge, exploring the concept of odds, highlighting the differences between luck and skill, and creating more negative attitudes toward gambling. Furthermore, four studies found that such improvements were maintained in the long term [46,51,54,60]. A number of studies also reported improvements in such skills as coping (two of three studies that assessed it [46,48]), awareness and self-monitoring (100% of the studies that assessed it [36,43,48]), the dialogue about gambling with other students and family members [43], problem-solving and decision-making [49]. Furthermore, the reviewed gambling prevention programs generally reported (84% of the studies that assessed these variables) good results with regard to a reduction in the frequency and severity of gambling [34,36,38,45,49,50,52,58,59,61,62]. However, only five of them explored the maintenance of such improvements in the long term, as is the study by Neighbors et al. (2015) [58], finding that all intervention effects except reduced gambling problems remained at the 6-month follow-up.

### 3.3. Program Content and Implementation

#### 3.3.1. Content

Most of the programs involved dynamic and interactive sessions (90.6%; the only ones that did not were those carried out with college students [42,45,58]), and were held in school hours and at the actual educational facility. Their main aims were to raise awareness in young people of the consequences that gambling can have, correct possible biases, develop different skills, and encourage critical thought and self-control. They typically made use of debates, presentations, testimonials, and video projections, among others. In this regard, a number of studies [47,57] reported the use of audiovisual aids to be conducive to attracting the attention of younger population groups, increasing the effects of interventions and, consequently, preventing and/or reducing gambling activity. Special mention should be made of the study by Ren, Moberg, and Scuffham (2019) [43], which offered activities that involved families, an essential point for preventing and dealing with gambling-related problems.

#### 3.3.2. Number of Sessions

In general, the number of sessions comprising the analyzed programs ranged between three and six, with a duration of approximately 70 min and containing different activities. There were exceptions, however, such as the study by Chóliz, Marcos, and Bueno (2021) [38], which consisted of two sessions. There was also one program that included an optional reinforcement session [49].

#### 3.3.3. Format or Method of Implementation

All the programs included in this systematic review were implemented in an educational setting, which meant that their implementation had to adapt to the conditions of the facility and available classrooms. They combined digital and interactive resources with participative activities, such as group debates. An online program featured in only one study [52], and five programs were facilitated by teachers [44,50,55,56,65].

### 3.4. Measurement Instruments Used

In general, the measurement instruments used were scales that assessed knowledge, attitudes, biases regarding gambling and specific skills, and ad hoc scales and questionnaires to rate gambling frequency, gambling-related risk, and problem gambling, such as the South Oaks Gambling Screen (SOGS), Gambling Quantity and Perceived Norms Scale (GQPN), and Problem Gambling Severity Index (PGSI), among others. Most of the studies did not include information on the problem/at-risk gamblers in their sample, nor did they make comparisons of results based on this variable.

### 3.5. Study Quality

The overall quality of the 32 included studies was rated as fair. Only nine (28%) included studies (9/27 controlled studies) were rated as good, while 11 (34%) (7/27 controlled studies and 4/5 single-arm studies) were rated as poor. For controlled studies, the risk of bias was mainly due to lack of ITT analysis or other unsuitable statistical analysis, the lack of blinding and sample size/power calculation, high dropout rates at the endpoint (>20%), or the differences between groups at baseline. For single-arm studies, the risk of bias was related to failure to prespecify and clearly describe the eligibility/selection criteria for the study population, lack of sample size calculation, lack of follow-up measures, and lack of information on blinding and sample representativeness.

## 4. Discussion

The efficacy of the programs included in this systematic review remains somewhat unclear given important methodological flaws that include problems with measurement, high variability between types of instruments and results, and short-term follow-up assessments. Although most of the studies did not include long-term results, the short-term benefits of these prevention programs highlight improvement in knowledge, a reduction in gambling frequency, and fewer erroneous notions about gambling among adolescents and young adults [41,46,65,66]. However, when follow-up assessments are not performed, there is no way of knowing whether gambling behaviors are really affected in the long term. It would be preferable for studies to assess behavior-related results over a period of six months or longer because there seems to be evidence that effects are lost over time [41,43,46,51]. Moreover, measurements of gambling-related problems and harm should reflect follow-up periods: SOGS-RA and DSM-IV-J/MR-J refer to the last 12 months; however, a number of studies simply adjusted these measurements to adapt them to shorter assessment periods [48,51,52,60], which is not appropriate. Furthermore, some studies did not provide an adequate classification or gambler types using the SOGS-RA [51,52]. In relation to the amount of money wagered/lost, it is difficult in this population group to detect reductions given that the amounts that tend be involved are not high; in addition, many of the instruments were self-report methods, with the consequent limitation of the reliability of the data. It is necessary to highlight the importance of using objective and validated measurement instruments for results that are appropriate to the aims of each study or intervention.

Moreover, special attention must be given to the content of programs and the way in which they are implemented. Only a few were designed according to evidence-based principles [67] or underwent systematic testing. In the case of studies conducted in the university setting, most used personalized normative feedback (PNF), with positive results as a preventive strategy for harm reduction; however, they may have undesirable consequences when addressing occasional gamblers by producing a boomerang effect [68]. What is more, programs in only a few studies involving adolescents were facilitated by teachers, despite the advantages, particularly in terms of feasibility and cost-effectiveness that this may bring. Although Ladouceur et al. (2003) [56] reported that the initiatives facilitated by gambling experts were more effective for reducing cognitive errors in students compared to those facilitated by their teachers, more recent studies [55,65] appear to produce highly positive results with this implementation format, although they stress that teachers should receive specific gambling-related training in order to achieve the desired effects [55]. Therefore, more studies of this type should be conducted in order to allow more solid conclusions to be drawn in this respect. In this regard, online programs or modules may also be considered promising resources, with positive results obtained in the few studies conducted in this format, all with adolescents [40,41,52,57]. Moreover, the use of digital methods may facilitate the development of preventive strategies for the young population group in general, and because the anonymity of participants is safeguarded, it may also be a recruitment strategy of interest.

It should be highlighted that most of the studies focused on adolescents, with a limited number of gambling prevention programs for the young adult group. In addition, all the studies involving university students were conducted in the United States and Canada, with an extraordinary absence of European studies. Bearing in mind that gambling habits can vary according to cultural and social contexts, and matters of a political or legal nature [2], this probably limits the ability for results to be generalized. Consequently, studies should be conducted to assess the effectiveness of prevention programs on young adults in other countries. Additionally, all the studies were conducted in educational settings, high schools and universities, most likely due to the accessibility and availability of this population group. Nonetheless, not including other subsamples of young adults could limit the generalization of conclusions to the general population. It has been demonstrated that gambling affects all kinds of people [16], with both high and low education and socioeconomic levels, which leads us to believe that it is necessary to apply interventions that address all settings, not only education. Therefore, the need to broaden gambling prevention programs to other youth population groups remains unresolved.

### 4.1. Recommendations

Based on the available evidence, we believe that gambling prevention programs should mainly be applied in a universal manner and as early as possible, although without neglecting the use of these strategies in young adults to prevent them from developing erroneous ideas regarding gambling, and to change any existing ones. As far as possible, they should be implemented in one or more sessions, even with the addition of reinforcement/reminder sessions, and long-term follow-up of changes is required [43]. It may be of interest to develop stepped programs that focus on teaching mathematical principles, and progressing from the simplest to the most complex so as not to overwhelm young people. It is essential that programs are relevant to young people in terms of their application and content; in other words, using new technologies and appealing resources, with multimedia elements and examples, and taking into account the social aspect of gambling and families in the process. Finally, assessments should measure both reductions and the harm caused, as well as gambling behaviors.

### 4.2. Strengths and Limitations

The main strengths of this systematic review are that the article searches were performed in scientific databases and trial registries, and the selection decisions were verified by three reviewers. It is also noteworthy that the criteria included RCTs, quasi-experimental trials, and trials with pre–post analyses, so the risk of not including studies relevant to the objective of the review was lowered.

However, there are certain limitations in the present review that should be taken into account. First, due to the variability of the methods in which the different variables of interest (such as gambling frequency) were registered and the heterogeneity of how the results achieved in them were reported, it has not been possible to perform a meta-analysis. Moreover, it should be noted that the frequency and amount of money gambled, among other data, were mostly self-reported, with the inevitable associated bias. Second, only nine studies (out of 32) concluded that the risk of bias was low, so it is necessary to state that higher-quality studies are needed. Third, data on long-term follow-up assessments (6 or more months of follow-up) were only recorded in seven studies [45,46,49,49,51,55,58,61], with different outcome variables under study, which made it difficult to draw conclusions about the trend toward the maintenance of changes after the interventions were completed. Finally, only studies published in English and Spanish were included, which may have implied the loss of articles relevant to the objective of the review.

## 5. Conclusions

The outlook for gambling is continuously changing as a result of new technologies, new forms of gambling, social acceptance, and greater ease of access, which is cause for increasing concern. Youth gambling is an important public health problem that must be addressed; however, researchers and clinicians have yet to develop a set of best practices for the prevention of gambling. More longitudinal and evidence-based studies are required that are designed on a solid theoretical base, with recruitment for and implementation of programs in settings other than education. In addition, more studies should be conducted in Europe and Spain, particularly among young adults. We believe the incorporation of youth gambling into the framework of public health [69], using a multidimensional perspective that acknowledges individual and social determining factors and draws on the principle of health promotion is a suitable approach to enable these problems in the young population to be tackled. There is still a great deal of work to be done, particularly with regard to prevention, which is only now beginning to gain attention.

## Figures and Tables

**Table 1:** Characteristics of the studies included in the review.

Study	Participants ^a^	Type of Prevention	Intervention	Comparison	Follow-Ups ^b^	Result Measurements (Instrument)	Results
**RCT design**							
Ferland et al. (2002) [47] Canada	N = 424 Age (M = 13.1) (Range 11–15) 46.7% female % PGs = ns	Universal	“Lucky” video + 40 min of information. 20 min video. Facilitator: Psychologist specializing in pathological gambling. Resource: video.	-Reading/activities. -“Lucky” video. -Control. -All conditions were decided by a psychologist specializing in pathological gambling.	-	-Ad hoc questionnaire divided into three scales: general knowledge, knowledge of excessive gambling and stereotypes.	The three experimental conditions were significantly more effective than the control for improving erroneous concepts (significant effects for Group (F(3, 416) = 15.86, *p* < .001), Time (F(1, 416) = 119.08, *p* < .001), and Interaction (F(3, 416) = 8.56, *p* < .001)) and knowledge (significant effects for Group (F(3, 416) = 12.90, *p* < .001), Time (F(1, 416) = 194.43, *p* < .001), and Interaction (F(3, 416) = 8.86, *p* < .001)).
Turner et al. (2008) [48] Canada	N = 201 Age (M = 16.5, SD = 1291) (Range 15–18) 67.16% female % PGs = 3.5%	Universal	Six 70 min sessions. Facilitator: Not reported. Use: Lessons, overhead projections, text and one CD-ROM, and questions for debate.	Control	-	-The gambling problem was measured with the South-Oaks Problem Gambling Screen-Revised for Adolescents (SOGS-RA). -Coping skills were assessed with the Preventive Resources Inventory (PRI). -Knowledge of random events (true/false test). -Awareness of problem gambling and self-monitoring (4-point Likert scale).	The experimental group showed a significant improvement in awareness of random events [intergroup difference F(1, 9.4) = 14.7, *p* < .01; the difference for the experimental group was 11.5% (95% CI = 8.6–14.4%), while the difference for the control group was 1.1% (95% CI = −1.8–4.0%)], self-control skills [F(1, 8.4) = 6.4, *p* < .05; the mean difference score for the experimental group was 6.1% (95% CI = 2.1–10.2%); the control group had a mean change score of −0.1% (95% CI = −4.1–3.9%)], and knowledge of coping skills [F(1, 9.6) = 9.7, *p* < .02); the experimental group increased by 3.4% (95% CI = 0.5–6.4%); the control group showed −2.3% (95% CI = −5.2−0.6%)] in comparison to the control group. With regard to knowledge about random events (d = 1.13), self-control (d = 0.34), and coping (d = 0.18), the program had an impact between strong and low for students who most needed the information.
Williams et al. (2010) [49] Canada	N = 949 Age (M = 16.0; SD = 1.0) (range 14–20) 47% female % PGs = 3.2% (DSM-IV-MR-J)	Universal	“Stacked Deck” program. Five basic lessons (standard program) + 1 optional lesson (reinforcement program). Face-to-face in the classroom. Facilitator: Trained research assistants. Resources: PowerPoint presentation, video, games.	Control	2: 3–7 months	-Attitudes toward gambling. -Scale of knowledge about gambling. -Scale of fallacies about gambling. -Decision-making and problem-solving skills. -Participation in high-risk activities. -Gambling behaviors. -DSM-IV-Multiple Response-Juvenile.	The students in the intervention groups had significantly more negative attitudes toward gambling [F(2, 1235) = 15.4, *p* < .001)], improved their knowledge about gambling [F(2, 1235) = 35.1, *p* < .001], improved their resistance to fallacies about gambling [F(2, 1235) = 34.4, *p* < .001], improved decision-making and problem-solving [F(2, 1235) = 6.29, *p* = .002], and reduced their gambling frequency [F(2, 1235) = 4.07, *p* = .017] and the percentage of gamblers (*p* < .001). There were no changes in participation in high-risk activities or the money lost to gambling.
Wohl et al. (2013) [35]	N = 72 Age (M = 19.69; SD = 1.82) (range 18–28) 70.2% female % PGs = ns (DSM-IV)	Universal	Animated video + reminders about preestablished betting limits. The 9 min video was designed to examine erroneous concepts about how electronic gambling machines work and to correct any erroneous concepts in a cognitively simple way.	Nine-minute video from the Ontario Lottery and Gaming Corporation explaining the annual revenues from lotteries, lottery games, and the location of different casinos in Ontario.	-	-Problem Gambling Severity Index. -Cognitive distortions (25-item Informational Biases Scale). -Betting limit detection (ad hoc item). -Adherence to preestablished betting limit (ad hoc item).	The results showed that both the video and reminders helped gamblers to keep within the preestablished monetary limit [X^2^ (1) = 6.83, *p* = .009 and X^2^ (1) = 6.83, *p* = .009, respectively]. Among participants who were not given reminders, those who watched the video kept within their preestablished monetary limits (94.1%) more than those who did not watch the video (61.1%), X^2^(1) = 5.40, *p* = .02.
Walther et al. (2013) [50] Germany	N = 2109 Age (M = 12; SD = 0.85) (range = 10–15) 49.6% female % PGs = ns	Universal	“Vernetzte” Four 90 min lessons. Facilitator: previously trained teachers. Resource: activities.	Control	-	-Lifetime gambling behaviors and frequency (ad hoc questions generated by the research team). -Attitudes and beliefs regarding betting games (selection of 4 items from the Gambling Attitudes and Beliefs Scale). -Knowledge about gambling (ad hoc scale developed by the research team).	The results of the multilevel fixed-effects regression analyses revealed significant effects of the program in terms of increased knowledge about gambling (d = 0.18), a reduction in problem gambling attitudes (d = 0.15), and a reduction in actual gambling (d = 0.02) in the intervention group in comparison to the control group. The program had no significant effect on lifetime gambling.
Todirita and Lupu (2013) [40] Romania	N = 81 Age (M = 12.5; SD = 0.707) Range (12–13) 54.32% female % PGs = ns	Universal	“Amazing Chateau” CD-ROM. Ten sessions, one per week. Facilitator: gambling expert. Resource: software.	-Control. -“Rational Emotive Education” (REE) program.	-	-Ad hoc questionnaire on gambling to gather information about knowledge, erroneous concepts, illusion of control, attitudes, cognitive errors.	The experimental group that received the “Amazing Chateau” program significantly improved their accuracy in the gambling-related questionnaire compared to the REE and control groups (F(2, 78) = 21.97, *p* = .000). However, the REE group was significantly better than the control group (*p* = .001).
Donati et al. (2014) [51] Italy	N = 181 Age (M = 15.95; SD = 1.29) (range 15–18) 36% female % PGs = ns	Universal	Facilitator: Not reported. Use: Activities with random event generators, PowerPoint presentation, video, and group debates. Each teaching module included exercises in which students had to apply the learned skill/concept.	Control	6 months	-The gambling problem was measured with the South-Oaks Problem Gambling Screen-Revised for Adolescents (SOGS-RA). -Correct knowledge and erroneous concepts about gambling (Questionnaire of Attitudes and Knowledge About Gambling). -Ability for probabilistic reasoning (Gambler’s Fallacy Task). -Perception of economic advantages of gambling (Gambling Attitude Scale). -Superstitious thinking (Superstitious Thinking Scale).	The results showed that the intervention was effective for improving knowledge about gambling (F(1, 145) = 12.62, *p* < .01, η^2^ = 0.08) and reducing erroneous concepts (F(1, 145) = 10.84, *p* < .01, η^2^ = 0.07), perception of the economic advantages of gambling (F(1, 143) = 7.16, *p* < .01, η^2^ = 0.05), and superstitious thinking (F(1, 141) = 5.48, *p* < .05, η^2^ = 0.04). There was no significant effect for normative probabilistic reasoning (F(1, 145) = 0.05, *p* = .83). Effects were achieved both in participants classified as no-problem gamblers and those classified as at-risk/problem gamblers at the start of the intervention. The training effects remained steady over time (6-month follow-up).
Canale et al. (2016) [52] Italy	N = 168 Age (M = 15.01; SD = 0.60) Range (14–18) 42% female % PGs = 8.3% (SOGS-RA)	Universal	Online intervention comprising 5 sessions. Facilitated over a 3-week period. Facilitator: Computer. Resource: online activities.	Control	2 months	-Gambling behavior was measured with the South-Oaks Gambling Screen-Revised for Adolescents (SOGS-RA). -Gambling attitudes (Gambling Attitudes Scale) (GAD).	There was a significant reduction in problem gambling in the intervention group at follow-up compared to the control group (d = 0.23). Frequent gamblers in the intervention group reduced problem gambling (d = 0.41) and frequency (d = 0.45) compared to occasional gamblers.
Huic et al. (2017) [53] Croatia	N = 190 Age (M = 15.61; SD = 1.29) (range 14–17) 32.4% female % PGs = ns	Universal	“Who really wins?” Six 9 min units of learning, facilitated in the classroom during school hours. Facilitator: two experts in working with adolescents and pathological gambling. Resources: questions, games and group activities, such as role-playing exercises.	Control	-	-Knowledge about games of chance/betting games (ad hoc questionnaire). -Cognitive distortions regarding gambling (Gambling-Related Cognitive Beliefs Scale). -Skills for resisting peer pressure (ad hoc questionnaire). -General self-efficacy (Generalised Self-Efficacy Scale). -Gambling behavior (ad hoc questionnaire). -Problem gambling (Canadian Adolescent Gambling Inventory).	The program significantly improved students’ knowledge about the consequences of gambling (F(1, 135) = 33.703, MSE = 4.640, *p* < .001, d = 0.89), dispelled gambling-related myths (F(1, 140) = 5.795, MSE = 0.302, *p* < .05, d = 0.46), and reduced cognitive distortions of all types (F(1, 141) = 4.748, MSE = 0.236, *p* < .05, d = 0.28). However, the program did not lead to any significant changes in intra- and interpersonal skills (self-efficacy, problem-solving, resistance to peer pressure) or gambling frequency after its implementation.
Calado et al. (2019) [54] Portugal	N = 111 Age (M = 17.64; SD = 1.62) 65% female % PGs = ns	Universal	Five 1 h units of learning aimed at increasing correct knowledge and reducing erroneous ideas about gambling, and to study the risk factors associated with gambling behaviors. Facilitator: Researchers.	Control	6 weeks	-Questionnaire on erroneous ideas and knowledge about gambling. -Gambling attitudes (Attitudes Towards Gambling Scale). -Sensation seeking (Brief Sensation Seeking Scale). -DSM-IV-Multiple Response-Juvenile (DSM-IV-JMR).	Compared to the control group, the experimental group showed significant improvement in terms of knowledge about gambling (F(1, 109) = 48.91, *p* < .001, ηp2 = 0.31) and reducing erroneous knowledge about gambling (F(1, 109) = 59.55, *p* < .001, ηp2 = 0.35), in addition to gambling attitudes (F(1, 109) = 14.91, *p* < .001, ηp2 = 0.12) and total hours per week spent gambling (F(1, 109) = 4.8, *p* < .05, ηp2 = 0.04). The number of at-risk/problem gamblers was also reduced during the study period. The results remained steady during the follow-up period.
Lloret-Irles and Cabrera-Perona (2019) [39] Spain	N = 330 Age (M = 15.7; SD = 0.67) (range 15–17) 59% female % PGs = ns	Universal	“¿Qué te juegas?” [What’s at stake?] Based on the Theory of Planned Behavior (TPB). Three 50 min sessions. Facilitator: Six experts in gambling prevention. Resources: presentation, debate, group dynamics, viewing and commentary on cases.	Control	-	-Three questions on intention to gamble. -Seven-item self-report questionnaire on self-efficacy for not betting. -Risk perception: risk perception subscale of the Early Detection Gambling Addiction Risk—Adolescents (EDGAR-A) scale. -Illusion of control and ignorance of gambling probability scale. -Gambling Advertising Attitude scale.	Students in the experimental group showed a significant reduction in intention to gamble (t = 3.156; d = 0.16), risk perception (t = 2.933; d = 0.21), favorable attitude to gambling advertising (t = 5.736; d = 0.33), and erroneous beliefs regarding probability (t = 7.063; d = 0.49). Self-efficacy for not betting increased (t = −3.922; d = 0.24). No pre–post changes were reported in the control group.
Tani et al. (2021) [55] Italy	-Teachers Experimental: N = 15 M = 52.27; SD = 5.93) (range = ns) Control: N = 18 M = 50.22; SD = 9.99) (range = ns) -Students Experimental: N = 219 M = 16.2; SD = 1.40) (range = 13–19) Control: N = 174 M = 16.57; SD = 1.36) (range = 13–19) % PGs = ns	Universal	Prevention program with a 16 h duration divided into 4 modules. Facilitator: Trained vs. untrained teachers. Resources: PowerPoint presentation, debates, guided practical exercises.	Control	7 months	Students: -Student gambling behavior: South Oaks Gambling Screen-revised for Adolescents (SOGS-RA). -Gambling-related cognitions (Gambling Related Cognitions Scale). -Gambling attitudes (Gambling Attitudes Scale). Teachers: -Gambling-related beliefs and knowledge (ad hoc questionnaire).	The results showed that the teachers in the experimental group improved their knowledge on the types and characteristics of games of chance/betting games and the related risks. The most important result was the impact that trained teachers had on their students, who reduced their gambling behavior (F(1, 300) = 8.40, *p* = .003, η^2^ = 0.029), some cognitive distortions [predictive control (F(1, 300) = 27.02, *p* = .000, η^2^ = 0.083), illusion of control (F(1 ,300) = 4.85, *p* = .023, η^2^ = 0.016) and gambling expectations (F(1, 300) = 4.15, *p* = .042, η^2^ = 0.014)], and erroneous concepts regarding economic advantages of gambling [lack of economic advantages (F(1, 299) = 4.74, *p* = .030, η^2^ = 0.016), and economic advantages (F(1, 299) = 4.85, *p* = .028, η^2^ = 0.016)].
Ladouceur et al. (2003) [56] Canada	-Stage 1: N = 153 (M = ns, SD= ns) 44% female -Stage 2: N = 356 (M = ns, SD= ns) 51% female % PGs = ns	Selective	“Count Me Out” Program consisting of one 1 h session that comprised exercises to be resolved on pathological gambling. -Group 1: C-T: Exercises from the “Count Me Out” program. Facilitator: Teacher. -Group 2: C-S: Exercises from the “Count Me Out” program. Facilitator: gambling expert. -Group 3: three exercises were created Facilitator: gambling psychology expert.	Group E-S: Exercises facilitated by a gambling expert.	-	Understanding of odds and randomness (Gambling Questionnaire).	The exercises facilitated by a gambling expert were more effective (F(2, 134) = 6.63; *p* < .05) for reducing erroneous perception than the exercises facilitated by the teacher (*p* < .05) and the gambling psychology expert (*p* < .05).
Ladouceur et al. (2004) [57] Canada	N = 371 Age (M = 12.8; SD = 0.7) (range 12–15) 48.2% female % PGs = ns	Selective	“Lucky” A 20 min video explaining the differences between betting games and games of skill, and aspects related to dangerous gambling. Resource: video.	Control	-	Ad hoc questionnaire to assess knowledge and erroneous concepts about pathological gambling.	The experimental group shown the video significantly improved their knowledge (F(1, 368) = 7.723, *p* < .01; η^2^ = 0.021; power = 0.79) and erroneous concepts (F(1, 368) = 15.772, *p* < .001; η^2^ = 0.041; power = 0.98) in post-intervention assessment compared to the control group.
Lupu and Lupu (2013) [41] Romania	N = 75 Age (M = 13.5, SD = 0.707) (Range 12–13) 52% female % PGs = ns	Selective	“Amazing Chateau” (AC) + “Rational Emotive Education” (REE). Ten sessions, one per week AC facilitator: gambling expert. REE facilitator: a psychologist and psychiatrist specializing in pathological gambling. Resource: software.	-Control. -“Rational Emotive Education” (REE).	-	Ad hoc questionnaire on gambling to gather information about knowledge, erroneous concepts, illusion of control, attitudes, cognitive errors.	The results obtained showed that the REE + AC group gave significantly more correct answers compared to the REE group alone and the control group (F(2, 78) = 21.97, *p* = .000), and those results remained steady throughout all the follow-up assessments performed.
Celio and Lisman (2014) [34] United States	N = 136 Age (M = 25.5; SD = 5.339) Range (17–34) 45% female % PGs = ns	Selective	“Personalized normative feedback”. Brief, one-session intervention that causes a behavioral change by focusing on and correcting misperceptions of what is “normal” or “typical” behavior. Facilitator: Computer.	-Control (data on students enrolled at the university).	1 week	-Measure of risk-taking behavior (Balloon Analogue Risk Task––BART). -Measure of gambling (Pick-A-Card (PAC) task). -Gambling frequency (Gambling Quantity and Perceived Norms scale).	After one week, the experimental group was found to have strongly reduced their perception of other students’ gambling (F(1, 132) = 39.25, *p* < .001, η^2^ = 0.23), perceived annual spending (F(1, 132) = 32.38, *p* < .001, η^2^ = 0.20), and maximum amount lost in one day (F(1, 132) = 18.78, *p* < .001, η^2^ = 0.12), unlike participants in the control group. The participants in the experimental group also took fewer risks in the two measured gambling analogies (BART and PAC), compared to the control group.
Neighbors et al. (2015) [58] United States	N = 252 Age (M = 23.11; SD = 5.34) 40.5% female % PGs = ns	Selective	“Personalized normative feedback”. The participants received feedback via a sheet of paper containing different types of gambling-related information. Facilitator: Research assistants.	-“Attention-control feedback”.	3 and 6 months	-Problem gambling (SOGS). -Problem gambling frequency and related aspects (Gambling Quantity and Perceived Norms scale). -Gambling-related problems (Gambling Problems Index).	At 3-month follow-up, significant effects of the intervention were found in the reduction of the real quantity lost (b = −0.506, t(224) = −2.79, d = 0.37, *p* = .005) and gambling-related problems (b = −0.720, t(224) = −2.42, d = 0.32, *p* = .016); reduction in perceived norms for quantities lost (b = −0.340, t(224) = −2.61, d = 0.35, *p* = .009) and won (b = 0.450, t(224) = −3.05, d = 0.41, *p* = .002). At 6-month follow-up, all effects of the intervention, except reduction in gambling-related problems, remained steady.
Williams (2002) [36] Canada	N = 597 Age (M = ns SD = 1) Range= 15–17 48.5% female % PGs = ns	Indicated	Program comprised of 5 sessions in which the following distinctions were made: 1. Information on the nature of gambling and pathological gambling. 2. Exercises to reduce cognitive errors regarding fallacies about gambling. 3. Information on odds in games of chance and their calculation. 4. Test of decision-making skills. 5. Test of adaptability and problem-solving skills. Facilitator: Not reported	Control	3 months	-Gambling behavior (DSM-IV-MR-J). -Coping strategies (modified version of Ways of Coping Checklist). -Ad hoc questionnaire on knowledge, awareness, attitudes, cognitive errors, and bet calculation in problem gambling.	The post-intervention assessment showed reductions in positive attitudes (t = −2.59, *p* < .001), better knowledge (t = −13.04, *p* < .001), and fewer cognitive errors (t = 8.23, *p* < .001), compared to the control group. At 3-month follow-up, the students in the intervention group showed significantly better knowledge (t = −8.87, *p* < .001), more negative attitudes (t = −6.65, *p* < .001), and fewer cognitive errors (t = 8.02, *p* < .001), compared to the control group. The intervention group was the only group to significantly reduce gambling frequency (F(2, 510) = 3.06, *p* < .05) and spending (F(2, 510) = 3.60, *p* = .014) during follow-up.
Takushi et al. (2004) [59] United States	N = 302 Age (M = ns; SD = ns) (range 18–21) % PGs = ns	Indicated	“Personalized feedback intervention” (PFI). One 45–60 min session in which participants received feedback from the assessment results. Facilitator: Not reported.	Control	3 months	-Gambling behavior (South Oaks Gambling Screen). -Gambling severity (Gambling Severity Index). -Aspects related to gambling behavior (Gambler’s Self-Report Inventory).	At 3-month follow-up, a reduction in gambling behavior (frequency and severity) was reported for both groups. The PFI group showed a greater reduction in the number of episodes of drinking and gambling at the same time.
Williams et al. (2004) [60] Canada	N = 578 Age (M = 16.2; SD = ns) (range ns) 47% female % PGs = 3.5% (DSM-IV-MR-J)	Indicated	“Gambling: A stacked deck”. Program consisting of five 75–100 min sessions, with information and exercises on the nature of gambling and gambling-related problems, and related aspects. Facilitator: Not reported. Resources: PowerPoint presentation.	Control	3 months	-Pathological gambling (DSM-IV-MR-J). -Gambling attitudes (Gambling Attitudes Scale). -Gambling knowledge (Gambling Knowledge Scale). -Gambling fallacies (Gambling Fallacies Scale). -Decision-making and problem-solving skills (Decision-Making and Problem-Solving Scale). -Dangerous gambling behaviors in the previous 3 months.	In the first study, the intervention group showed significant improvement in mathematical reasoning in gambling (F(2, 330) = 30.3, *p* < .001) and fallacies (F(2, 330) = 28.6, *p* < .001), compared to the control group at 6-month follow-up. In the second study, the intervention group showed significant improvement in knowledge (F(1, 548) = 38.5, *p* < .001), fallacies (F(1, 548) = 22.3, *p* < .001), and attitudes (F(1, 548) = 12.3, *p* < .001), and significant reductions in the time spent (F(1, 548) = 15.8, *p* < .001) and money spent gambling (F(1, 548) = 9.5, *p* < .01) compared to the control group at 3-month follow-up.
Hopper (2008) [42] United States	N = 68 Age (M = 21.40 SD = 4.27) Range= 18–44 10% female % PGs = ns	Indicated	“Personalized normative feedback”. The participants received feedback with different types of gambling-related information. Facilitator: Not reported.	Control	3 months	-Problem gambling (SOGS). -Readiness to change (Gambling Readiness to Change Scale). -Problem gambling frequency and related aspects (Gambling Quantity and Perceived Norms scale). -Gambling-related problems (Gambling Problems Index).	The intervention group reported no changes at 1-month follow up regarding the amount and frequency of gambling behaviors, and willingness of participants to change their gambling behaviors, compared to the control group. Participants in the intervention group reduced their perceived norms about how much others gamble ((t (29) = 3.807, *p* = .001, d = 0.5), more than the control group.
Petry et al. (2009) [61] United States	-BA: N = 32 Age (M = 20.2; SD = 1.9) 21.9% female -MET: N = 30 Age (M = 20.5; SD = 1.4) 13.3% female -MET + CBT: N = 21 Age (M = 20.1; SD = 1.4) 9.5% female -Control: N = 34 Age (M = 20.5; SD = 2) 14.7% female % PGs = ns	Indicated	-“Brief advice” (BA): Immediately after assessment lasting 10–15 min. Facilitator: Therapist -“Motivation Enhancement therapy” (MET): Individual 50 min session after assessment Personalized comments were given about the student’s gambling behavior. Facilitator: Therapist -MET + cognitive behavioral therapy (CBT): An MET session was held after baseline assessment. Facilitator: Therapist. Participants were encouraged to return for three individual sessions of CBT.	Control	9 months	-Problem gambling (SOGS). -Gambling-related problems (National Opinion Research Center DSM-IV). -Addiction severity (Addiction Severity Index-Gambling section) (ASI-G). Assessments were performed at baseline, 6 weeks, and 9 months. -Gambling type and frequency (TimeLine Followback). -Services received regarding problem gambling (Treatment Service Review).	In comparison with the assessment-only group (control), participants receiving an intervention of some kind showed significant reductions in scores ASI-G (t(115) = 2.28, *p* < .05), days (t(115) = 2.24, *p* < .04), and dollars wagered (t(115) = 2.22, *p* < .05) over time. The MET + CBT groups showed benefits in some but not all gambling indices. None of the interventions differed significantly for these three variables. With regard to the control group, the MET group had a positive significant association, with an odds ratio (OR) of 3.41, indicating that those who received MET were three times more likely to be classified as “substantially improved” compared to the control group. Neither the BA nor MET + CBT groups significantly altered their likelihood of substantial improvement.
Larimer et al. (2012) [45] United States	N = 147 Age (M = 21.23; SD = 1.37) (range 19–25) 34.7% female % PGs = ns	Indicated	“Personalized feedback intervention” (PFI). One 60–90 min session in which participants received feedback from the assessment results. Facilitator: Therapists.	-Cognitive Behavioral Intervention (CBI): Six 60 min sessions. -Assessment-only control (AOC).	6 months	-Gambling behavior (South Oaks Gambling Screen). -Problem gambling frequency and related aspects (Gambling Quantity and Perceived Norms scale). -Gambling-related problems (Gambling Problems Index). -Pathological gambling (National Opinion Research Center DSM-IV Screen). -Illusion of control (Beliefs About Control Scale).	At 6-month follow-up, both the PFI and CBI groups were associated with a reduction in the consequences of gambling (*p* = .032; d = 0.48, and d = 0.,39, respectively) and the DSM-IV criteria (*p* = .006; d = 0.60, d = 0.48). The PFI group showed reductions in gambling frequency (d = 0.48) and perceived norms (d = 0.68), compared to the AOC group. Changes in perceived norms mediated the association between the PFI group and gambling frequency (Z = 2.01, *p* = .044). The CBI group showed reductions in illusion of control (d = 0.43).
Martens et al. (2015) [62] United States	-PFB: N = 111 Age (M = 21.69; SD = ns) Range (ns) 38% female -EDU: N = 113 Age (M = 22.19; SD = ns) Range (ns) 42% female -AO: N = 109 Age (M = 21.84; SD = ns) Range (ns) 42% female % PGs = ns	Indicated	“Personalized feedback only intervention” (PFB). The participants received feedback via a sheet of paper containing different types of gambling-related information. Facilitator: Not reported.	-Only education (EDU). -Assessment only (AO).	3 months	-Problem gambling (SOGS-RA). -Problem gambling frequency. -Pathological gambling-related problems (Problem Gambling Index).	At 3-month follow-up, participants in the PFB group reported less money wagered (X^2^ = 4.95, *p* = .03, d = 0.25) and fewer gambling-related problems (X^2^ = 8,43, *p* < .03, d = 0.32) than those in the AO group. No differences were found between PFB and EDU groups.
**Quasi-experimental design**							
Gaboury et al. (1993) [46] Canada	N = 289 Age (M = 16, SD: ns) (Range: ns) % PGs = ns	Universal	Three 75 min sessions held over a 3-week period. Facilitator: Not reported. Resources: videos and questionnaires.	Control	6 months	Questionnaire to assess participants’ gambling behaviors. Diagnostic criteria made use of the South Oaks Gambling Screen (SOGS).	The program improved knowledge about gambling (F(2, 5) = 23.5, *p* < .05), which remained steady at 6-month follow-up, and coping skills (F(2, 5) = 67.7, *p* < .01) in the experimental groups vs. the control group. The variance analyses performed on gambling behaviors and attitudes were not significant. The program was rated interesting or very interesting by 77% of the participating students. For 52%, the most informative aspect of the program was that “gambling can become excessive or pathological and cause severe problems” and that “gambling can turn into an illness or an addiction.”
Davis (2002) [63] Canada	N = 452 Age (M = 15.4; SD = 0.76) Range (14–19) 49% female % PGs = ns	Universal	The program was 5 sessions long, taught in five consecutive class periods, with each session having a duration of 80–100 min. Facilitator: Researcher. Resources: PowerPoint presentation, video, exercises.	Control	3 months	-Awareness and knowledge of problem gambling. -Attitudes toward gambling. -Susceptibility to cognitive errors underlying gambling fallacies. -Ability to recognize and calculate true gambling odds. -Coping strategies (modified version of Ways of Coping Checklist). -Gambling-related problems (Gambling Problems Index). -Problem gambling (DSM-IV-Multiple Response-Juvenile).	The results showed significant prevention for knowledge of gambling (F(2, 542) = 34.01, *p* < .001), gambling attitudes (F(2, 542) = 6,14, *p* < .01), cognitive errors (F(2, 542) = 11.21, *p* < .001), and calculation of gambling-related odds (F(2, 542)—7.97, *p* < .001). However, no significant change was found for the gambling behavior of the experimental group compared to the control group.
Ladouceur et al. (2005) [64] Canada	N = 568 Age (M = 15.99; SD = 0.79) (range ns) 53% female % PGs = ns	Selective	“Gambling Stories”. 20 min video. Facilitator: Research assistant. Resource: video	Control	1 month	Ad hoc questionnaire divided into three scales: general knowledge, knowledge of excessive gambling and stereotypes.	The video significantly increased general knowledge about gambling (F(1, 491) = 33.03; *p* < .0001) and knowledge about excessive gambling (F(1, 493) = 18.06; *p* < .0001), and reduced stereotypes (F(1, 481) = 24.36; *p* < .0001).
**Pre- and post-test design**							
Taylor and Hillyard (2009) [44] United States	N = 8455 Age (M = ns; SD = ns) (range ns) 52% female % PGs: 10% (MSOGST)	Indicated	“Don’t Gamble Away our Future”. Program with a 45 min duration comprising reading, activities, and discussion exercises. Facilitator: Trained professionals. Resources: CD-ROM.	-	-	Knowledge about pathological gambling (scale designed by the Illinois Institute for Addiction Recovery).	Knowledge about games of chance and their negative effects improved significantly after the intervention (t (8.454) = −50.89, *p* = .000), compared to baseline assessment.
Ren et al. (2019) [43] United States	2 groups: -N = 16,421 (with MSOGST test) -N = 16,262 (without MSOGST test) Age (M = 13; SD = 3.317) Range (8–18) % PGs = ns	Universal	“Don’t Gamble Away our Future” (DGAOF). Fourteen 45–60 min sessions facilitates throughout one academic year. Students could take part in the program a number of times, but in different years. Facilitator: Researcher. Resources: PowerPoint presentation, CD-ROM.	-	-	-Ad hoc questionnaire on pathological gambling-related knowledge. -Pathological gambling behaviors (Modified South Oaks Gambling Screen for Teens; only students in the 5th grade and higher).	The students who received multiple interventions obtained higher scores in the pre-test, compared to students who received a single intervention (*p* < .001 for all comparisons between the groups), and showed a tendency to increased awareness of gambling over time (*p* < .001 for multiple interventions; *p* = .538 for the single intervention). The prevalence of problem gambling had decreased among students receiving the intervention twice as compared to receiving the intervention once (7.9% vs. 9.4%; OR = 0.89, 95% CL: 0.82–0.97). However, this effect was not confirmed among students who received the intervention three or more times.
Berrios et al. (2020) [37] Spain	N = 637 Age (M = ns; SD = ns) 46.8% female % PGs = ns	Universal	“Cubilete” [“Dice Cup”]. Program consisting of four 50 min sessions held in the course of one month. Facilitator: Psychologists specializing in addictions and obsessive-compulsive disorders with the presence and collaboration of the homeroom teacher. Resources: discussions, videos, group activities.	-	-	-Beliefs about, attitudes to, and use of technology, virtual gaming, and gambling (ad hoc questionnaire).	The results showed that after facilitation of the program, there was a reduction in use of technologies, online gaming, and gambling, and in smartphone use (χ2 = 333.23, *p* < .05) and costs associated with smartphone use (χ2 = 116.57, *p* < .05).
Chóliz et al. (2021) [38] Spain	N = 2,372 Age (M = 16.5; SD = 1.871) (Range 14–19) 48.8% female % PGs = ns	Universal	2 sessions Facilitator: Psychologist specializing in addictions and pathological gambling. Resources: audiovisual aids such as charts, graphs, news articles, testimonials from pathological gamblers and patients, and advertisements.	-	1 month	-Questionnaire on gambling patterns. -Assessment scale to assess both frequency of traditional forms of gambling and online gambling.	After the prevention program, the percentage of adolescents who gambled every month significantly decreased (traditional gambling: X^2^_1_ = 99.26, *p* < .001, Phi = 0.15; online gambling: X^2^_1_= 39.93, *p* < .001, Phi = 0.10), as did the percentages of adolescents who presented with behaviors corresponding to dangerous gambling (X^2^_1_= 41,43, *p* < .001, Phi = 0,10) and gambling disorder (X^2^_1_= 6.17, *p* < .01, Phi = 0.04).
Dodig et al. (2021) [65] Croatia	N = 629 Age (M = 15.67; SD = 0.73) (range ns) 33.5% female % PGs = ns	Universal	“Who really wins?” Facilitator: School counselors and teachers. Program consisting of nine 45 min sessions with students, plus a 2 h interactive session with parents and another with members of the school.	-	-	-Gambling behavior (ad hoc questionnaire). -Gambling-related knowledge (ad hoc questionnaire). -Cognitive distortions regarding gambling (Gambling-Related Cognitive Beliefs Scale). -Problem-solving skills (ad hoc questionnaire). -Skills for resisting peer pressure (ad hoc questionnaire). -General self-efficacy (Generalised Self-Efficacy Scale). -Problem gambling severity (Problem Gambling Severity Scale).	The results show that the program is effective for reducing gambling-related cognitive distortions (illusion of control: t = 12.15, *p* < .001, d = 0,48; probabilistic reasoning and superstition: t = 9.14, *p* < .001, d = 0.36) and improving knowledge of gambling (t = 14.63, *p* < .001, d = 0.58); however, it is not effective for improving social and emotional skills (problem-solving skills, coping with peer pressure skills, and general self-efficacy).

^a^ Percentage of problem gamblers at baseline. ^b^ Months between intervention completion and follow-up assessment.

## Data Availability

Not applicable.

## Supplementary Materials

The following supporting information can be downloaded at: https://www.mdpi.com/article/10.3390/ijerph20064691/s1, Table S1: Risk of bias for controlled intervention studies based on the Heart, Lung, and Blood Institute assessment tool; Table S2: Risk of bias for single-arm studies based on the Heart, Lung, and Blood Institute assessment tool.
