# Supplementary material for: Preventive Gambling Programs for Adolescents and Young Adults: A Systematic Review
Source: Int J Environ Res Public Health. 2023 Mar 7;20(6):4691. doi: 10.3390/ijerph20064691 (PMC10048743; doi:10.3390/ijerph20064691)
Supplement: Supplementary file 1 [file ijerph-20-04691-s001.zip › ijerph-2247469-supplementary.pdf]

Supplementary Data

Table S1: Risk of bias for controlled intervention studies based on the Heart, Lung, and Blood Institute assessment tool

Study	1	2	3	4	5	6	7	8	9	10	11	12	13	14	QA
Ferland et al. (2002) [47]	Y	Y	Y	N	NR	Y	Y	Y	Y	NR	N	N	Y	Y	Good
Turner et al. (2008) [48]	Y	N	N	N	NR	Y	Y	Y	NR	NR	Y	N	Y	Y	Fair
Williams et al. (2010) [49]	Y	N	Y	N	NR	Y	N	Y	NR	NR	Y	N	N	NR	Fair
Wohl et al. (2013) [35]	Y	NR	NR	NR	Y	Y	Y	Y	Y	NR	Y	N	Y	Y	Good
Walther et al. (2013) [50]	Y	Y	Y	N	NR	N	Y	Y	Y	NR	N	Y	Y	N	Fair
Todirita and Lupu (2013) [40]	Y	NR	CD	N	NR	Y	Y	Y	Y	NR	N	N	Y	Y	Good
Donati et al. (2014) [51]	Y	Y	Y	N	NR	Y	N	Y	NR	NR	Y	N	Y	N	Fair
Canale et al. (2016) [52]	Y	Y	Y	N	NA	Y	N	CD	NR	NR	Y	N	Y	N	Poor
Huic et al. (2017) [53]	Y	Y	Y	N	NR	Y	Y	N	NR	NR	Y	N	Y	N	Fair
Calado et al. (2019) [54]	Y	Y	Y	N	NR	Y	Y	Y	NR	NR	Y	N	Y	Y	Good
Lloret-Irles and Cabrera-Perona (2019) [39]	Y	NR	NR	N	NR	Y	Y	Y	NR	NR	Y	N	Y	Y	Good
Tani et al. (2021) [55]	Y	Y	CD	N	NR	Y	N	Y	Y	NR	Y	N	Y	NR	Fair
Ladouceur et al. (2003) [56]	Y	Y	CD	N	NR	N	NR	NR	Y	NR	Y	N	CD	N	Poor
Ladouceur et al. (2004) [57]	Y	Y	NR	N	NR	Y	N	NR	Y	NR	N	Y	N	N	Poor
Lupu & Lupu (2013) [41]	Y	Y	NR	N	NR	Y	Y	Y	Y	NR	N	N	CD	N	Fair

Celio and Lisman (2014) [34]	Y	Y	Y	N	NR	Y	Y	NR	Y	NR	Y	N	Y	Y	Good
Neighbors et al. (2015) [58]	Y	Y	Y	Y	NR	Y	Y	Y	Y	NR	Y	N	Y	Y	Good
Williams (2002) [36]	Y	Y	Y	N	NR	N	Y	Y	Y	NR	N	Y	Y	N	Poor
Takushi et al. (2004) [59]	Y	NR	Y	Y	N	NR	Y	CD	Y	NR	Y	N	N	N	Poor
Williams et al. (2004) [60]	Y	NR	CD	N	N	Y	Y	NR	NR	NR	Y	N	Y	NR	Fair
Hopper (2008) [42]	Y	NR	CD	N	NR	Y	Y	NR	Y	NR	Y	Y	Y	N	Fair
Petry et al. (2009) [61]	Y	Y	Y	N	NR	CD	Y	Y	Y	NR	Y	Y	N	Y	Good
Larimer et al. (2012) [45]	Y	Y	N	N	NR	Y	N	Y	Y	NR	Y	N	N	Y	Fair
Martens et al. (2015) [62]	Y	NR	CD	N	NR	Y	Y	Y	Y	NR	Y	Y	Y	Y	Good
Gaboury et al. (1993) [46]	Y	N	NR	NR	NR	NR	NR	NR	Y	NR	Y	N	N	N	Poor
Davis (2002) [63]	Y	Y	Y	N	NR	N	Y	Y	Y	NR	N	N	Y	NR	Fair
Ladouceur et al. (2005) [64]	N	N	Y	N	NR	N	NR	NR	Y	NR	N	N	N	NR	Poor

Note: CD, cannot determine; NA, not applicable; NR, not reported

List of risk of bias categories:

1.- Was the study described as randomized, a randomized trial, a randomized clinical trial, or an RCT?
2.- Was the method of randomization adequate (i.e., use of randomly generated assignment)?
3.- Was the treatment allocation concealed (so that assignments could not be predicted)?
4.- Were study participants and providers blinded to treatment group assignment?
5.- Were the people assessing the outcomes blinded to the participants' group assignments?
6.- Were the groups similar at baseline on important characteristics that could affect outcomes (e.g., demographics, risk factors, co-morbid conditions)?
7.- Was the overall drop-out rate from the study at endpoint 20% or lower of the number allocated to treatment?
8.- Was the differential drop-out rate (between treatment groups) at endpoint 15 percentage points or lower?
9.- Was there high adherence to the intervention protocols for each treatment group?
10.- Were other interventions avoided or similar in the groups (e.g., similar background treatments)?
11.- Were outcomes assessed using valid and reliable measures, implemented consistently across all study participants?
12.- Did the authors report that the sample size was sufficiently large to be able to detect a difference in the main outcome between groups with at least 80% power?
13.- Were outcomes reported or subgroups analyzed prespecified (i.e., identified before analyses were conducted)?
14.- Were all randomized participants analyzed in the group to which they were originally assigned, i.e., did they use an intention-to-treat analysis?

The tool and its items can be found here: https://www.nhlbi.nih.gov/health-topics/study-quality-assessment-tools

Table S2: Risk of bias for before-after (Pre-Post) studies with no control group based on the Heart, Lung, and Blood Institute assessment tool

	1	2	3	4	5	6	7	8	9	10	11	12	QA
Taylor & Hillyard (2009) [44]	Y	N	CD	CD	N	N	Y	NR	Y	Y	N	NA	Poor
Ren et al. (2019) [43]	Y	N	Y	CD	Y	Y	Y	NR	CD	Y	Y	NA	Fair
Berrios et al. (2020) [37]	Y	N	Y	CD	N	Y	Y	NR	CD	Y	N	NA	Poor
Chóliz et al. (2021) [38]	Y	N	CD	CD	NR	Y	Y	NR	NR	Y	N	NA	Poor
Dodig et al. (2021) [65]	Y	N	CD	CD	NR	Y	Y	NR	NR	Y	N	NA	Poor

Note: CD, cannot determine; NA, not applicable; NR, not reported.

List of risk of bias categories:

1.- Was the study question or objective clearly stated?
2.- Were eligibility/selection criteria for the study population prespecified and clearly described?
3.- Were the participants in the study representative of those who would be eligible for the test/service/intervention in the general or clinical population of interest?
4.- Were all eligible participants that met the prespecified entry criteria enrolled?
5.- Was the sample size sufficiently large to provide confidence in the findings?
6.- Was the test/service/intervention clearly described and delivered consistently across the study population?
7.- Were the outcome measures prespecified, clearly defined, valid, reliable, and assessed consistently across all study participants?
8.- Were the people assessing the outcomes blinded to the participants' exposures/interventions?
9.- Was the loss to follow-up after baseline 20% or less? Were those lost to follow-up accounted for in the analysis?
10.- Did the statistical methods examine changes in outcome measures from before to after the intervention? Were statistical tests done that provided p values for the pre-to-post changes?
11.- Were outcome measures of interest taken multiple times before the intervention and multiple times after the intervention (i.e., did they use an interrupted time-series design)?
12.- If the intervention was conducted at a group level (e.g., a whole hospital, a community, etc.) did the statistical analysis take into account the use of individual-level data to determine effects at the group level?

The tool and its items can be found here: https://www.nhlbi.nih.gov/health-topics/study-quality-assessment-tools
